# Periodontitis and Tooth Loss Have Negative Systemic Impact on Circulating Progenitor Cell Levels: A Clinical Study

**DOI:** 10.3390/genes10121022

**Published:** 2019-12-07

**Authors:** Gaetano Isola, Antonino Lo Giudice, Alessandro Polizzi, Angela Alibrandi, Romeo Patini, Sebastiano Ferlito

**Affiliations:** 1 Department of General Surgery and Surgical-Medical Specialties, School of Dentistry, University of Catania, Via S. Sofia 78, 95123 Catania, Italy, nino.logiudice@gmail.com (A.L.G.); alexpoli345@gmail.com (A.P.);; 2 Department of Biomedical, Odontostomatological Sciences and of Morphological and Functional Images, School of Dentistry, University of Messina, 98100 Messina, Italy; 3 Department of Economical, Business and Environmental Sciences and Quantitative Methods, University of Messina, Piazza Pugliatti 1, 98100 Messina, Italy; 4 Fondazione Policlinico Universitario Agostino Gemelli IRCCS, Institute of Dentistry and Maxillofacial Surgery, Università Cattolica del Sacro Cuore, 00168 Rome, Italy; romeo.patini@unicatt.it

**Keywords:** periodontal health, diet, genes, CD133+, KDR+, periodontitis, circulating progenitor cells, stem cells, cardiovascular disease

## Abstract

The aim of the present study was to investigate the association and impact of periodontitis and tooth loss on a subtype of endothelial progenitor cell (EPC) levels (CD133^+^/KDR^+^). Furthermore, the objective was to determine if the periodontal status influenced CD133^+^/KDR^+^ levels. In all, 88 patients with periodontitis and 79 healthy controls (HCs) were enrolled in the study. Enrolled patients were examined and characterized by clinical and blood sample analysis. Spearman’s correlation test was applied in order to assess the interdependence between CD133^+^/KDR^+^ levels and all periodontal parameters. In order to estimate a statistically significant trend (*p*-trend) for ordered CD133++/KDR+ quartiles, the Jonckheere–Terpstra test was applied for all variables. Patients in the periodontitis group presented significantly lower CD133^+^/KDR^+^ levels (66.4 (45.5–269.6 cells/µL)) compared to the HC group (76.7 (24.3–313.2 cells/µL), *p* < 0.001). Lower CD133^+^/KDR^+^ levels negatively correlated with C-reactive protein (CRP), with the number of teeth, and with all periodontal parameters (*p* < 0.001). Moreover, there was a proportional increase in CD133+/KDR+ levels with a progressive increase in number of teeth (*p*-trend < 0.001), while there was a proportional decrease in CD133+/KDR+ levels with a proportional increase in clinical attachment level (CAL, *p*-trend = 0.003), probing depth (PD, *p*-trend = 0.007), and bleeding sites (bleeding on probing (BOP), *p*-trend < 0.001) as an extent measure of periodontitis. This study demonstrated that patients with periodontitis presented significantly lower CD133^+^/KDR^+^ levels compared to HCs. Moreover, all patients presented an increase in the CD133^+^/KDR^+^ EPC levels with an extended level of periodontitis and tooth loss.

## 1. Introduction

Periodontitis is an inflammatory disease associated with a microbial dysbiosis, which may determine the destruction of the supporting tissues of the teeth and alveolar bone resorption through a specific elicited inflammatory host response [1]. Periodontitis has been broadly associated with different systemic conditions such as cardiovascular disease (CVD) [2], diabetes [3], and endothelial dysfunction [4,5,6]. 

During the last few decades, several studies have revealed a close link between periodontitis and CVD. More specifically, Higashi et al. have shown that periodontal disease, in subjects without risk factors for CVD, is a condition associated with a high dysfunction of the endothelium and blood vessels, through a decrease in nitric oxyde (NO) production, and that systemic inflammation may be one of the leading causes of CVD [6,7,8].

Recently, a key role played in the etiology of periodontitis has been highlighted by a subtype of stem cells derived from bone marrow, the circulating endothelial progenitor cells (EPCs). EPCs possess the ability to express surface antigens of endothelial and hematopoietic stem cells and to assist in maintaining vascular integrity and the repair mechanism of the endothelium [9]. Among the primary markers for the analysis of EPC levels are CD34^+^, CD133^+^, and the kinase insert domain-containing receptor (KDR). CD34^+^ and CD133^+^ originate from hematopoietic stem cell antigens, whereas KDR is a specific marker of endothelial cells [10]. More specifically, CD34^+^ and CD133^+^/ KDR^+^ allow less mature and mature EPCs to be evaluated [11]. 

The number and functionality of EPCs have been proposed as markers for prodromal endothelial damage correlated to the CVD event and, as such, a method to assess the ability to repair vascular damage following a CVD incident [12]. Recently, the number of EPCs was validated as one of the primary subclinical markers linked directly to increased CVD risk [11,13].

However, studies that analyzed the association between periodontitis and impaired EPCs reported conflicting results. In some studies, periodontitis was correlated with high levels of EPCs [14,15], whereas some other studies reported a limited association between periodontitis and impaired EPCs, even after adjusting for important confounders [16,17]. However, these studies did not specifically report any association between the extent of periodontitis and CD133^+^/KDR^+^ levels and the *p*-trend of this association.

On this basis, the aim of this preliminary cross-sectional study was to investigate a possible association between a subtype of EPCs, CD 133^+^, and the extent of periodontitis. Furthermore, the objectives were to determine whether periodontal parameters significantly had an impact on EPCs and if periodontal parameters changed with EPC increase.

## 2. Materials and Methods

### 2.1. Study Design

The present study was designed as a cross-sectional study. A total of 332 patients, matched for age and gender, were initially enrolled from February 2016 to November 2018 among those who attended the Department of Periodontology, School of Dentistry of the University of Messina, Messina, Italy. The local ethical committee approved the study protocol (#16-012, march 2016). All patients were informed about the characteristics of the study and provided their informed written consent. The study was performed following the guidelines of the Declaration of the World Medical Association 1975 in Helsinki, revised in 2000. The study was reviewed and checked following the STROBE (Strengthening the reporting of observational studies in epidemiology) guidelines (see Supplementary Materials 1).

### 2.2. Patients

The inclusion criteria for all patients were as follows: (1) aged >18 years and (2) already enrolled for dental treatment. 

Subjects with a diagnosis of periodontitis [18] were enrolled for the periodontitis group. Inclusion criteria for the periodontitis group were as follows: (1) presence of at least 16 teeth; (2) a minimum of 40% of sites with a clinical attachment level (CAL) ≥2 mm and probing depth (PD) ≥4 mm [19,20] ; (3) presence of at least ≥2 mm of crestal alveolar bone loss verified on digital periapical radiographs; (4) presence of ≥40% sites with bleeding on probing (BOP) [21,22]; and (5) no systemic diseases. By considering the new classification of periodontal disease [18], periodontitis patients could be classified as stage 1, grade B, generalized periodontitis patients.

Healthy individuals presented no systemic disease and no sites with PD ≥4 mm or CAL ≥4 mm or radiographic signs of bone loss.

The exclusion criteria for all patients were as follows: (1) intake of contraceptives; (2) intake of antibiotics, immunosuppressive drugs, or anti-inflammatory drugs throughout the last three months prior to the study; (3) status of pregnancy or lactation; (4) previous history of excessive drinking; (5) allergy to local anesthetic; (6) intake of drugs that may potentially determine gingival hyperplasias such as hydantoin, nifedipine, cyclosporin A, or similar drugs; (7) periodontal therapy throughout the last three months prior to the study; and (8) presence of systemic diseases.

The demographic (level of education), clinical and medical characteristics (sex, age, body mass index (BMI)) [23], and medication were assessed in all enrolled subjects. BMI was estimated on the weight of the patient divided by the square of the patient’s height (i.e., kg/m^2^). Periodontitis patients with self-reported diabetes at baseline were excluded from this analysis.

Using a cross-sectional design, after the first screening, 165 patients were excluded from the final sample because they did not meet the inclusion criteria (*n* = 123), declined to participate (*n* = 26), or missed the first appointment (*n* = 16). Thus, for this study, a total of 157 patients, 88 patients with periodontitis and 79 healthy subjects, were finally enrolled.

### 2.3. Clinical Data: Periodontal Examination and Collection

The periodontal evaluation comprised the recording of PD, CAL, plaque index (PI) [20,24], and BOP, the latter being evaluated during PD assessment by the presence of bleeding up to 30 seconds after probing. CAL was recorded as PD plus recession with the cementoenamel junction as a reference for CAL measurements [19,25]. All clinical periodontal parameters were recorded at six sites per tooth on all teeth present excluding third molars. Moreover, patients were asked the main cause of tooth loss.

All clinical periodontal parameters were recorded, in all patients, at six sites per tooth on all teeth present excluding third molars by two independent calibrated examiners (a principal examiner and a second control examiner) not involved in the subsequent data analysis with a manual periodontal probe (UNC-15, Hu-Friedy, Chicago, IL, USA). In the case of discordant measurements ≥2 mm for CAL, a new clinical assessment was carried out. The inter-examiner reliability test resulted in an agreement of 86.5% (*k* = 0.63) for the outcome CAL. The intra-examiner agreement was evaluated by the measurement of Cohen’s k coefficient, which was 0.828, and which equaled a high degree of reliability.

### 2.4. Power and Sample Size

The sample size was established considering a number of groups equal to 2, an effect size of 0.5 for CD133^+^/KDR^+^ count (that represents the primary periodontal variable), an expected standard deviation of 0.5 [14], a two-sided significance level of 0.05, and a power of 80%. It was determined that approximately 53 patients per group would be needed. The enrolled patients were about 80 per group, so that the achieved power value was 91%.

### 2.5. Laboratory Analyses

During the first clinical examination, all patients underwent venous blood sampling at 8:30 a.m. Extensive chemical analyses were performed at the medical center after overnight fasting in all subjects. Glucose, plasma lipids, and fibrinogen were determined by routine laboratory methods analysis. C-reactive protein (CRP) levels are expressed as milligrams per liters (mg/L) and were obtained by a commercially available enzyme-linked immunoassay (ELISA) kit (Sigma-Aldrich, Milan, Italy). 

The circulating EPC levels were detected by the analysis of the expression of surface markers CD133^+^/KDR^+^ and measured by activated fluorescence analysis of cells as previously described [26]. More specifically, a 100 mL portion of peripheral blood was incubated with CD133 antibodies (Beckman Coulter, Fullerton, CA, USA).

### 2.6. Statistical Analysis

For each of the two groups, numerical data are expressed as mean ± SD or median and interquartile range (IQR) and for categorical variables as number and percentage. Examined variables did not present normal distribution as verified by the Kolmogorov–Smirnov test and, consequently, a non-parametric approach was used.

A baseline comparison between groups was performed using the unpaired *t*-test. Both for all groups and then for every single group of patients, the non-parametric Spearman’s correlation test was applied in order to assess the existence of significant interdependence between CD133^+^/KDR^+^ levels and all periodontal parameters. For each periodontal parameter (number of teeth, CAL, PD, BOP, and PI), a stepwise multivariable linear regression model was estimated in order to assess the dependence of each of them by potentially explicable variables such as age, sex, BMI, CD133^+^/KDR^+^ levels, total cholesterol, HDL-cholesterol, LDL-cholesterol, and CRP.

Quartiles of CD133^+^/KDR^+^ levels were calculated; thus, for each CD133+/KDR+ quartile, mean ± SD was calculated of all periodontal parameters. In order to estimate a *p*-trend for ordered CD133^+^/KDR^+^ quartiles, the Jonckheere–Terpstra test was applied for all variables. More specifically, we assessed whether the periodontal parameters significantly increased or decreased with a CD133^+^/KDR^+^ increase. Statistical analyses were performed using Statistical Package for Social Science SPSS (IBM, Bologna, Italy) version 17.0 for the Windows package. Furthermore, *p* < 0.05 (two-sided) was considered to be statistically significant.

## 3. Results

### 3.1. Study Participants

Sociodemographic variables of the study participants are presented in Table 1. All enrolled subjects were matched for age (*p* = 0.076), gender (*p* = 0.126), percentage of smokers (*p* = 0.321), and BMI (*p* = 0.075) (Table 1).

Patients in the periodontitis group presented significantly lower median values of CD133^+^/KDR^+^ levels (66.4 (45.5–269.6 cells/µL)) compared to patients in the healthy control (HC) group (79.7 (24.3–313.2 cells/µL), *p* < 0.001). 

In addition, patients in the periodontitis group showed higher median CRP levels (0.41 (0.35; 0.49)) compared to healthy controls (0.33; (0.27; 0.35), *p* < 0.001) (Table 1). 

The mean values of the periodontal parameters are presented in Table 2. The main cause of tooth loss in all enrolled patients was attributable to the presence of periodontitis. Patients in the periodontitis group presented a significantly lower number of teeth and higher PD, CAL, and BOP compared to healthy control subjects (*p* < 0.001), whereas there were no differences in the mean values of PI among the groups (*p* = 0.478).

### 3.2. Periodontal Status and CD133^+^/KDR^+^ Levels

**Figure 1:**
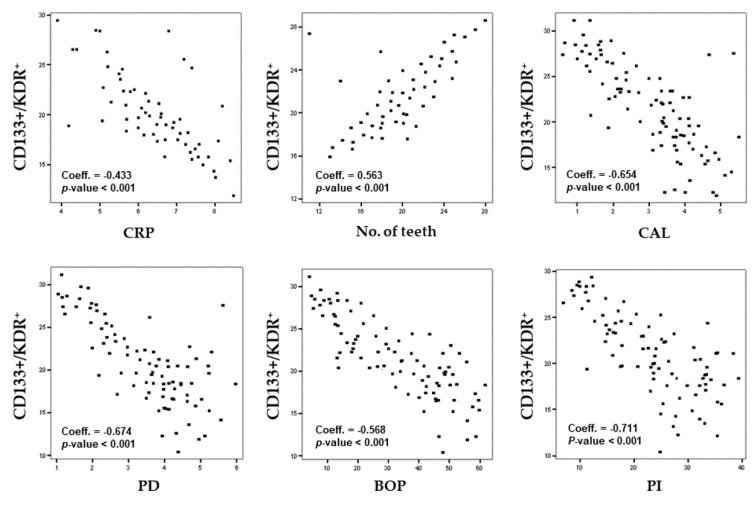
Spearman’s correlation between CD133^+^/KDR^+^ levels, C-reactive protein (CRP), number of teeth, and periodontal parameters.

Spearman’s correlation test performed for the whole sample showed that CD133+/KDR+ levels were negatively correlated with CRP levels (Coeff. = −0.433, *p* < 0.001), CAL (Coeff. = −0.654, *p* < 0.001), PD (Coeff. = −0.674, *p* < 0.001), BOP (Coeff. = −0.568, *p* < 0.001), and PI (Coeff. = −0.711, *p* < 0.001), and positively correlated with the number of teeth (Coeff. = 0.563, *p* < 0.001) (Figure 1).

Further univariate and multivariate stepwise regression analyses were performed to assess the possible associations between CD133^+^/KDR^+^ levels and the different features of periodontal health status in the whole sample. 

The results of the univariate regression models highlighted that, in all enrolled patients (periodontitis and healthy controls), CD133^+^/KDR^+^ levels significantly influenced the number of teeth and mean sites with BOP positive (*p* < 0.001). Table 3 shows the results of multivariate regression analysis. For each periodontal parameter, we report the regression coefficient, the 95% confidence interval (CI), and also the relative *p*-value.

In the stepwise multivariate regression model, for the whole sample (periodontitis and healthy controls), all potential confounding variables (age, gender, BMI, and CRP) were inserted. The results showed that the number of teeth, PD, CAL, and BOP were significantly related to CD133^+^/KDR^+^ levels (Table 3). 

In addition, in the whole sample, the number of teeth was significantly dependent also on age (*p* = 0.016), female gender (*p* = 0.009), CD133^+^/KDR^+^ (*p* < 0.001), CRP (*p* < 0.001), and HDL-cholesterol levels (*p* = 0.028). The CAL was significantly dependent on CD133^+^/KDR^+^ levels (*p* < 0.001), age (*p* = 0.026), and CRP levels (*p* < 0.001). The PD was significantly dependent on CD133^+^/KDR^+^ (*p* < 0.001) and CRP levels (*p* < 0.001), whereas the BOP was significantly dependent on CD133^+^/KDR^+^ (*p* < 0.001) and CRP levels (*p* < 0.001) (Table 3).

**Figure 2:**
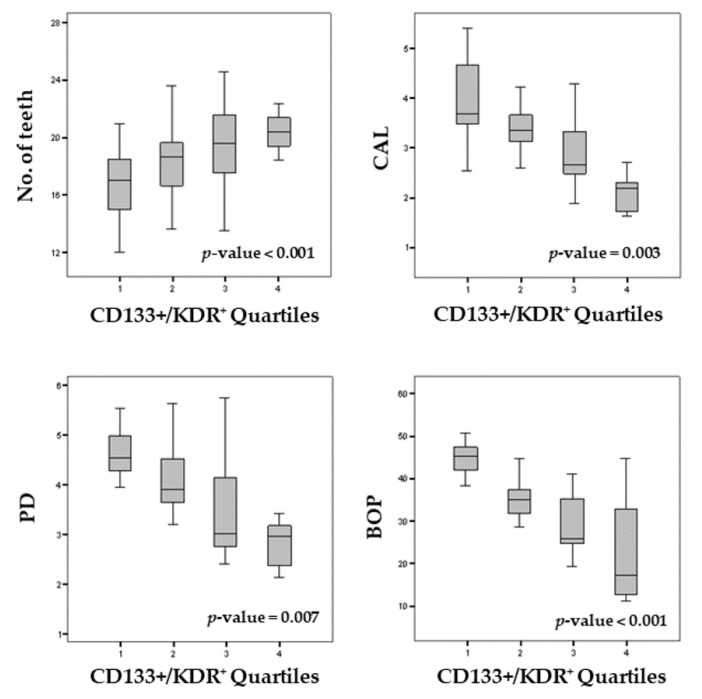
Boxplot of periodontal parameters according to CD133^+^/KDR^+^ quartiles and *p*-trend. Each *p*-trend value referred to an increase/decrease of periodontal parameters according to CD133+/KDR+ quartiles and was obtained by means of the Jonckheere–Terpstra test for ordered alternative hypothesis.

Moreover, in order to assess whether the periodontal parameters significantly increase or decrease as CD133^+^/KDR^+^ levels increase (quartiles), a *p*-trend for an ordered alternative hypothesis was estimated. The results obtained from the Jonckheere–Terpstra test showed that, in all patients, there was a proportional increase in CD133+/KDR+ levels with a progressive increase in number of teeth (*p*-trend < 0.001), while there was a proportional decrease in CD133+/KDR+ levels with a proportional increase in clinical attachment level (CAL, *p*-trend = 0.003), probing depth (PD, *p*-trend = 0.007), and bleeding sites (bleeding on probing (BOP), *p*-trend < 0.001) (Figure 2).

## 4. Discussion

This cross-sectional study analyzed the association between CD133^+^/KDR^+^ levels and periodontitis. The objectives were also to evaluate, in the same groups of patients, whether periodontal parameters and tooth loss were dependent on CD133^+^/KDR^+^ levels and whether periodontal parameters significantly increased or decreased with CD133^+^/KDR^+^ decrease.

The results of the present study showed that patients with periodontitis presented a significantly lower median proportion of CD133^+^/KDR^+^ levels (*p* < 0.001) compared to healthy controls. 

In accordance with our results, previous pilot studies that have analyzed the relationship between EPCs and periodontitis found impaired levels of EPCs in patients with periodontitis [16,17].

Periodontitis is known to be one of the main risk factors of several diseases, such as diabetes, CVD events, and endothelial dysfunction [27,28,29]. Moura et al. [30] evaluated, in a cross-sectional study, 47 patients with and without periodontitis and found that patients with periodontitis presented a significantly higher endothelial dysfunction risk than individuals without periodontitis.

During the last decades, a two-directional, reciprocal relationship between impaired EPC levels and periodontitis has been shown in some studies [14,16,31,32,33]. Li et al. [15] demonstrated that patients who presented moderate to severe periodontitis exhibited an increased risk of high EPC count, compared with those with no or mild periodontitis. More specifically, the authors found lower levels of CD34^+^ and KDR^+^ in patients with moderate to severe periodontitis and an inverse association with high CRP levels and EPCs, supporting the evidence of a two-way relationship between periodontitis and CVD.

The present study found that patients with periodontitis showed higher median CRP levels compared to healthy controls. A possible explanation of the high proportion of CRP levels in patients with periodontitis compared to healthy controls could be due to the strong impact exerted by periodontopathogenic bacteria on systemic inflammation mediated by a CRP pathway [34,35,36], mainly measured by CRP and interleukin 6 (IL-6). Similarly, several studies have shown that periodontal treatment could significantly determine a reduction in the levels of CRP [37,38,39,40]. 

In agreement with these reports, the results of the present study suggest the hypothesis that periodontitis may have led to an increase in the production of high levels of CRP perhaps as a condition of local and systemic stimulation of oxidative stress. The relatively high production of CRP could consequently stimulate an impairment of CD133^+^/KDR^+^ levels to protect cells from tissue damage due to oxidative stress-mediated by NO. In this regard, further studies aimed at analyzing NO, CRP, and EPC levels could clarify this important issue that could define the role played by impaired EPCs, such as one of the risk factors of CVD.

Moreover, the results of the present cross-sectional study showed also that lower CD133^+^/KDR^+^ levels negatively impacted the number of teeth and gingival bleeding (BOP). In accordance with our results, findings from some pilot studies have suggested that periodontitis may be negatively associated with EPCs [14,15,41]. 

Li et al. [15] identified EPCs through flow cytometry of peripheral blood mononuclear cells as either double-positive CD34^+^ or CD309^+^ cells. Although those authors did not report a higher mean level of EPCs in patients with periodontitis compared to that in healthy controls for either of the above marker combinations, they observed a higher proportion of patients with periodontitis than healthy controls among the values of EPC counts (32% versus 9% based on the CD34^+^/CD309^+^ combination and 30% versus 13% based on the CD133^+^/CD309^+^ combination). This could be explained by the fact that EPC levels may have been influenced by the serum collection method and analyses used in different studies.

Based on these pivotal observations, the current study was designed in order to evaluate whether periodontal parameters and tooth loss significantly increased or decreased with a decrease in CD133^+^/KDR^+^ levels. 

The results of the present study showed that there was a significant decrease in CD133+/KDR+ in patients which presented high periodontal parameters (CAL, *p*-trend = 0.003; PD, *p*-trend = 0.007; BOP, *p*-trend < 0.001), whereas there was an increase in the CD133+/KDR+ levels when patients presented a high number of teeth (*p*-trend < 0.001).

Previous studies, which have shown how progressive worsening of periodontitis is associated with a high risk of CVD development, underlining the importance that periodontal treatment may positively affect circulating EPCs and peripheral vascular endothelial function during periodontitis [42,43,44,45].

In this regard, a study by Wang et al. [33] on 18 subjects with moderate to severe periodontitis showed that active periodontal treatment significantly reduced the gene expression of IL-6 (fold change of −1.88), and IL-8 (fold change of −1.51) in EPCs. Moreover, another study on 22 healthy adults with moderate to severe periodontitis who underwent complete mouth disinfection found that, at one-month follow-up, periodontal treatment resulted in significant improvements in periodontal pocketing, flow-mediated dilation, and serum IL-6, as well as a trend toward reduction in serum CRP [46]. Furthermore, several studies have reported that an intensive periodontal treatment resulted not only in a temporary increase in serum levels of CRP and IL-6, but also in a significant improvement of endothelial dysfunction indexes at six months after active periodontal therapy [47,48,49].

The results of the present study have, however, some limitations. One concerns the study design. The cross-sectional design does not allow for an analysis of the temporal association between CD133^+^/KDR^+^ levels and periodontitis that should be assessed only with a longitudinal observation.

Moreover, we did not assess the carotid intima−media thickness (c-IMT) and flow-mediated dilation of the brachial artery that could better evaluate the possible endothelial dysfunction in the analyzed sample. Furthermore, the median CRP values of patients in both groups of the present study were slightly higher compared to those in other similar studies [50,51,52,53,54]. These differences in median CRP values may be due to a systematic bias linked to the laboratory assay used in the present study and, therefore, should be carefully considered when interpreting results.

## 5. Conclusions

In conclusion, the results of the present study suggest that low serum CD133^+^/KDR^+^ levels appear to be negatively correlated with periodontitis and with the periodontal health status. Moreover, the presence of periodontitis appears as a condition that negatively influenced CD133^+^/KDR^+^ levels. In this regard, low serum CD133+/KDR+ levels during periodontitis appear to be linked with the possibility of developing future endothelial dysfunction and CVD risk.

However, this preliminary study demands further long-term clinical trials with large sample size and with a prospective design aimed at adopting strategies to accomplish a more definitive endpoint of treatment. The trials are strongly needed to achieve convincing evidence to determine, with greater accuracy, the effects of periodontitis on the EPC changes and CVD-related risk.

## Figures and Tables

**Table 1:** Descriptive statistics of clinical features of examined groups and comparison among them. Blood values are represented, such as median and interquartile range (IQR) (1st; 3rd). HbA1c, hemoglobin A1c; HDL, high-density lipoprotein; LDL, ligh-density lipoprotein; BUN, blood urea nitrogen; EPC, endothelial progenitor cells.

Clinical Features	Reference Values	Healthy Controls (*n* = 79)	Periodontitis (*n* = 88)	*p*-Value
Male, *n* (%)		45 (56.9)	49 (55.6)	0.126
Age, mean ± SD		51.9 ± 5.2	52.8 ± 4.1	0.076
Education level				
Primary school, *n* (%)		29 (36.7)	31 (35.2)	0.558
High school, *n* (%)		28 (35.4)	32 (36.3)	0.641
College or university, *n* (%)		22 (27.8)	25 (28.4)	0.589
BMI, kg/m^2^, mean ± SD		24.2 ± 4.1	23.9 ± 4.2	0.075
Smoker, *n* (%)		17 (21.5)	19 (21.6)	0.321
Current, *n* (%)		10 (12.6)	11 (12.5)	0.311
Never, *n* (%)		48 (60.7)	53 (60.2)	0.158
Past, *n* (%)		4 (5)	5 (5.6)	0.233
Glucose, mg/dL	65–110	96.4 (83.1; 104.5)	97.9 (91.4; 138.2)	0.356
HbA1c, mmol/mol	up to 40	35.3 (29.6; 38.6)	36.1 (28.8; 50.2)	0.078
Uric acid, mg/dL	1.9–8	2 (1.5; 2.6)	2.9 (1.8; 3.9)	<0.001
Albumin, g/L	35–50	36.9 (32.4; 38.8)	37.6 (35.2; 41.5)	0.388
Fibrinogen, mg/dL	150–400	278.5 (221.4; 279.3)	282.7 (266.4; 318.5)	0.554
Apolipoprotein A, mg/dL	>120–140	130.4 (122.2; 137.6)	133.6 (129.5; 138.2)	0.667
Total cholesterol, mg/dL	<200	172.3 (154.1: 184.5)	178.1 (155.1; 185.5)	0.564
HDL-cholesterol, mg/dL	<40–60	50.5 (47.2; 58.1)	52.9 (47.8; 56.2)	0.602
LDL-Cholesterol mg/dL	<100–130	112.1 (105.5; 122.1)	118.6 (110.9; 127.2)	0.555
BUN, mg/dL	7–30	27.6 (25.5; 30.1)	29.1 (24.5; 30.6)	0.369
CRP(C-reactive protein), mg/L	<0.8	3.3 (2.7; 3.5)	4.1 (3.5; 4.9)	<0.001
Systolic pressure, mm/hg	110–130	120.5 (112.6; 132.3)	123.6 (117.1; 134.6)	0.557
Diastolic pressure, mm/hg	70–85	81.9 (74.5; 85.4)	83.9 (79.1; 85.5)	0.058
Ferritin, ng/mL	12–300	77.3 (70.5; 81.4)	84.2 (73.4; 86.5)	0.056
Vitamin D, ng/mL	5–75	28.5 (25.7; 35.1)	27.2 (20.4; 31.2)	0.114
CD34^+^/KDR^+^ EPC (cells/µL)		162.1 (55.1–289.5)	141.0 (19.4–896.2)	0.049
Low count (no. (%))		75 (94.3)	59 (67)	0.042
High count (no. (%))		4 (5)	29 (32.9)	
CD133^+^/KDR^+^ EPC (cells/µL)		79.7 (24.1–156.4)	66.4 (45.5-169.6)	<0.001
Low count (no. (%))		76 (96.2)	55 (62.5)	0.056
High count (no. (%))		3 (3.8)	33 (37.5)	

**Table 2:** Descriptive statistics of periodontal parameters of examined groups and comparison among them. CAL, clinical attachment level; PD, probing depth; BOP, bleeding on probing; SD, standard deviation.

Periodontal Parameters	Healthy Controls (*n* = 79)	Periodontitis (*n* = 88)	*p*-Value
Number of teeth, no., mean ± SD	24.8 ± 1.6	16.5 ± 1.4	<0.001
CAL, mm, mean ± SD	1.52 ± 0.8	3.91 ± 0.5	<0.001
% of sites with CAL 4 to 5 mm, ± SD	−	38.4 ± 3.4	<0.001
% of sites with CAL ≥6 mm, ± SD	−	21.2 ± 2.5	<0.001
PD, mm, mean ± SD	1.52 ± 1.3	4.61 ± 0.7	<0.001
% of sites with PD 4 to 5 mm, ± SD	−	44.7 ± 4.4	<0.001
% of sites with PD ≥6 mm, ± SD	−	23.4 ± 4.4	<0.001
BOP, mean % ± SD	9.2 ± 9.7	45.7 ± 3.1	<0.001
Plaque index (PI), mean ± SD	0.71 ± 0.3	0.86 ± 0.5	0.511

**Table 3:** Stepwise (backward elimination) linear regression models for periodontal parameters. n.s., not significant. CRP, c-reactive protein; CAL, clinical attachment level; PD, probing depth; BOP, bleeding on probing.

	**Number of Teeth**			**CAL**		
Variables	Coeff.	95% CI	*p*-Value	Coeff.	95% CI	*p*-Value
Age	−0.15	−0.24; −0.04	0.016	0.03	0.02; 0.65	0.026
Gender	1.62	0.48; 2.81	0.009	−	−	*n.s.*
CD133^+^/KDR^+^	0.34	0.16; 0.41	<0.001	−0.11	−0.14; −0.65	<0.001
CRP	−2.49	−3.17; −1.81	<0.001	0.46	0.25; 0.59	<0.001
HDL-Cholesterol	0.01	0.01	0.028	−	−	*n.s.*
						
	**PD**			**BOP**		
Variables	Coeff.	95% CI	*p*-value	Coeff.	95% CI	*p*-value
Age	0.03	−0.02; 0.79	0.061	0.74	0.22; 1.36	0.129
CD133^+^/KDR^+^	−0.09	−0.14; −0.49	<0.001	−1.69	−2.25; −1.17	<0.001
CRP	0.42	0.23; 0.65	<0.001	8.48	5.37; 10.45	<0.001
HDL-Cholesterol	−0.12	−0.02; 0.28	0.074	−	−	*n.s.*

## Supplementary Materials

The following are available online at https://www.mdpi.com/2073-4425/10/12/1022/s1, Supplementary Materials 1: STROBE Statement—checklist.
